# Outcomes of Portal Vein Thrombosis in Smokers With and Without Cirrhosis and Predictors of Mortality: A Nationwide Assessment

**DOI:** 10.7759/cureus.37658

**Published:** 2023-04-16

**Authors:** Vikash Kumar, Dhir Gala, Mili Shah, Naresh Kumar, Vijay Reddy Gayam, Praneeth Bandaru, Arnold N Forlemu, Denzil Etienne, Vinaya Gadaputi

**Affiliations:** 1 Internal Medicine, The Brooklyn Hospital Center, Brooklyn, USA; 2 Internal Medicine, American University of the Caribbean School of Medicine, Sint Maarten, SXM; 3 Gastroenterology and Hepatology, The Brooklyn Hospital Center, Brooklyn, USA; 4 Gastroenterology and Hepatology, Blanchard Valley Health System, Findlay, USA

**Keywords:** morality, smoker, cirrhosis, acute portal vein thrombosis, portal vein thrombosis

## Abstract

Portal vein thrombosis (PVT) is a rare condition that can lead to numerous complications, like variceal bleeding, hepatic encephalopathy, and chronic liver disease. PVT has various etiologies, including liver disease, infections, and hyper-coagulable disorders. Cirrhosis, a chronic progressive liver condition characterized by liver fibrosis, is one of the risk factors for the development of PVT. Secondly, smoking also increases the risk of PVT. The aim of this study is to identify outcomes in patients with PVT who smoked with and without cirrhosis. This study was performed using the National Inpatient Sample (NIS) database for the years 2016, 2017, and 2018. The study identified 33,314 patients diagnosed with PVT who smoked, of which 14,991 had cirrhosis, and 18,323 did not have cirrhosis. Patients with PVT and cirrhosis had significantly higher in-hospital mortality, upper gastrointestinal bleeds, acute kidney injury, and peritonitis compared to patients without cirrhosis. The results of the study show that patients with PVT and cirrhosis who smoke have a higher risk of unfavorable outcomes.

## Introduction

Portal vein thrombosis (PVT) is a rare but potentially serious condition characterized by the formation of a blood clot in the portal vein [1]. Common presentations include abdominal pain, nausea, vomiting, ascites, liver dysfunction, and even potentially fatal conditions like variceal bleeding and hepatic encephalopathy [2]. Multiple conditions, such as hyper-coagulable disorders (for example anti-phospholipid syndrome and factor V Leiden mutation), liver disease, infections (for example pylephlebitis, appendicitis, and diverticulitis), trauma, and specific medications (for example birth control pills, hormone replacement therapy, tamoxifen, thalidomide, and erythropoietin), can result in PVT [3]. In order to manage PVT and avoid complications, early diagnosis, and treatment are essential. Anticoagulation therapy, clot removal procedures, and management of any underlying conditions may all be part of the course of treatment [4]. One risk factor for the development of PVT is the presence of cirrhosis [5]. Additionally, individuals who smoke are at a greater risk due to the endothelial cell damage brought on secondary to smoking [6].

Cirrhosis is a chronic and progressive liver disease characterized by the formation of scar tissue in the liver [7]. This can lead to liver dysfunction failure. It is a serious, possibly fatal condition with several etiologies, heavy alcohol use, viral hepatitis, and autoimmune disorders [8]. In its early stages, cirrhosis may not show any symptoms and frequently progresses slowly. But as the condition worsens, symptoms like exhaustion, abdominal pain, jaundice, and ascites could appear [9]. Cirrhosis can lead to infections, an elevated risk of liver cancer, bleeding from varices in the esophagus or stomach, and other complications [10]. Although there is no cure for cirrhosis, there are treatments to control the symptoms and slow the disease's progression, including prescription drugs, dietary changes, and, in extreme cases, liver transplantation. To prevent and manage this serious, potentially fatal condition, it is crucial to comprehend the causes, risk factors, symptoms, and treatments for cirrhosis.

We aim to determine outcomes in patients diagnosed with PVT who smoke with and without cirrhosis. 

## Materials and methods

Study data

In this retrospective analysis, we used data from the Healthcare Cost and Utilization Project's National Inpatient Sample (NIS), which is sponsored by the Agency for Healthcare Research and Quality (HCUP). With information on over 7 million hospitalizations (unweighted), NIS is the largest publicly accessible all-payer administrative database; when weighted, it represents about 35 million hospitalizations nationally. Data for specific patients, doctors, and hospitals are safeguarded while information on clinical and resource utilization is provided. In order to reflect hospital systems' adoption of the International Classification of Diseases, Tenth Edition, Clinical Modification/Procedure Coding System (ICD-10-CM/PCS), the NIS began using it in October 2015. Using the Agency for Healthcare Research and Quality sampling and weighting method, national estimates of the entire United States of America hospitalized population were calculated.

Study design

Patients with age older than 18 years with the primary diagnosis of acute PVT and smoking were identified through a retrospective analysis using the National Inpatient Sample database from 2016, 2017, and 2018 and the ICD-10 codes. We divided the entire study population into two groups: PVT with cirrhosis and PVT without cirrhosis, and then looked at all-cause in-patient mortality, hospital length of stay (LOS), and total costs. We used the Charlson Comorbidity Index, insurance status, hospital characteristics, and pertinent comorbidities as baseline characteristics for patients. The chi-square test and the t-test were used to compare categorical and continuous variables, respectively. Utilizing multivariate logistic and linear regression analyses, confounding variables were corrected.

Outcomes

In-hospital mortality from all causes and mortality predictors were the main outcomes of interest. Sepsis, acute kidney injury, and peritonitis were among the secondary outcomes. ICD-10-CM/PCS for each was used to determine the incidence of complications. We also looked at the typical hospital costs and LOS.

Study analysis

The complex survey design of the NIS database was taken into account as we carried out all statistical analyses using the suggested techniques. Continuous data are presented as mean with standard deviation and standard error, while categorical data are expressed as frequency and percentage. The Student's t-test was used to analyze continuous variables, while Pearson's Chi-square test was used to analyze categorical variables. The primary and secondary outcomes' unadjusted odds ratios were calculated using univariate logistic regression. The final model included a multivariable logistic regression to account for potential confounders. The two-sided p-value cutoff for statistical significance was 0.05. For data analysis, we used STATA® Version 17.0 software (StataCorp., College Station, TX, USA). The threshold for statistical significance was set at p 0.05. To produce national estimates, all analyses in our study were weighted using the provided discharge weights. The Consumer Price Index was used to adjust the cost of the hospitals for inflation (provided by the U.S. Department of Labor).

## Results

From 2016 to 2018, a total number of 33,314 patients with a diagnosis of PVT were identified. Of these, 14,991 were also diagnosed with cirrhosis, while 18,323 were not. The mean age was 61 years and 60 years, respectively. Patients with PVT and cirrhosis had significantly higher in-hospital mortality, upper gastrointestinal bleeds, acute kidney injury and peritonitis. In addition, there was also a significant difference regarding race, hospital region and academic institute, insurance, and chronic comorbidities between the two groups (Table 1).

**Table 1:** Patients demographics, insurance providers, hospital bed size, region and teaching status and patient comorbidities comparing the difference in PVT and cirrhosis with PVT alone. PVT: portal vein thrombosis

Patient Characteristics	With Cirrhosis (N=14,991)	Without Cirrhosis (N=18,323)	p-value
Age (mean)	61	60	
Female	29.6	38.3	0.000
Race			
Caucasian	66.6	72.3	0.000
African American	10.2	12.2	
Hispanic	15.4	9.0	
Asian	3.5	2.8	
Native American	1.0	0.3	
Others	3.1	3.0	
Insurance			
Medicare	50.2	46.2	0.000
Medicaid	21.1	15.81	
Private	25.1	34.1	
Others/Uninsured	3.4	3.8	
Bed size			
Small	12.8	14.3	0.25
Medium	21.2	21.6	
Large	65.5	64.5	
Hospital Region			
Northeast	19.2	22.1	0.32
Midwest	24.0	26.5	
South	31.4	30.8	
West	25.2	20.4	
Teaching hospital	83.8	81.8	0.03
Chronic comorbidity			
Hypertension	36.6	41.9	0.00
Diabetes mellitus	38.8	29.6	0.000
Chronic kidney disease	14.8	8.8	0.000
Chronic heart failure	8.2	6.8	0.03
Obesity	13.5	13.4	0.91
Dyslipidemia	20.5	30.3	0.00
Coronary artery disease	14.1	15.0	0.31

We compared the outcomes between those diagnosed with PVT alone and those with both PVT and cirrhosis. The group with PVT and cirrhosis had a significantly higher in-hospital mortality compared to those without cirrhosis (OR = 2.8 [2.40-3.630], p = 0.00). Similarly, the incidence of upper gastrointestinal bleeds was significantly higher in patients with PVT and cirrhosis (OR = 1.87 [1.48-2.37], p = 0.00). The PVT and cirrhosis group had a higher incidence of acute kidney injury (OR = 2.45 [2.18-2.76], p = 0.00) and peritonitis (OR = 5.00 [3.82-6.82], p = 0.00). The incidence of sepsis and ileus was not significantly different between the two groups (Table 2). 

**Table 2:** Prognostic outcomes comparing the difference in PVT and cirrhosis and PVT alone. PVT: portal vein thrombosis, GI: gastrointestinal, AKI: acute kidney injury

Frequency in %	With Cirrhosis (N=14,991)	Without Cirrhosis (N=18,323)	Odds Ratio and Confidence interval	p-value
In-hospital mortality	10.0	3.6	2.8 [2.40-3.630]	0.00
Upper GI bleeding	6.2	3.4	1.87 [1.48-2.37]	0.00
AKI	31.6	15.8	2.45 [2.18-2.76]	0.00
Peritonitis	7.1	1.4	5.00 [3.82-6.82]	0.00
Sepsis	6.2	5.7	1.09 [0.89-1.33]	0.395
Ileus	2.53	2.95	0.85 [0.62-1.15]	0.31

There was a higher incidence of cirrhosis among patients with PVT in Hispanic (15.4 vs 9.0), Asian (3.5 vs 2.8), and Native American (1.0 vs 0.3) patients compared to a diagnosis of PVT alone. Caucasians and African Americans on the other hand had a higher incidence of PVT diagnosis compared to both PVT and cirrhosis. Teaching hospitals and hospitals in the South and West regions had a higher incidence of combined PVT and cirrhosis. Trends also showed a higher incidence of PVT and cirrhosis for those with Medicare, and Medicaid as opposed to PVT alone. Lastly, chronic comorbidities including diabetes mellitus (38.8 vs 29.6), chronic heart failure (8.2 vs 6.8), chronic kidney disease (14.8 vs 8.8), and obesity (13.5 vs 13.4) were all associated with a higher incidence of PVT and cirrhosis compared to PVT alone.

## Discussion

The results of this study demonstrate that patients with PVT and cirrhosis have significantly worse outcomes compared to those without cirrhosis. The in-hospital mortality rate, as well as the frequency of upper gastrointestinal bleeds, acute kidney injury, and peritonitis, were all significantly higher in the PVT and cirrhosis group. Between the two groups, there was no significant difference in the incidence of sepsis or ileus. The study also found differences in demographic and clinical characteristics between the two groups. The higher prevalence of cirrhosis among Hispanic, Asian, and Native American PVT patients raises the possibility of a racial disparity and calls for further research. The study also discovered differences between the two groups in terms of hospital region, teaching status, and insurance status.

The study found that patients with PVT and cirrhosis had significantly higher in-hospital mortality rates compared to those with PVT alone. Previous meta-analysis reported increased odds of mortality in the first year in patients with PVT and cirrhosis (OR = 0.32; 95% CI = 0.14-0.75; p = .008) [11]. Similarly, a previous cohort study reported similar findings of increased mortality in patients with PVT and cirrhosis [12]. 

These results highlight the significance of early PVT diagnosis and treatment, especially in cirrhotic patients. In order to avoid complications and achieve better outcomes, it is essential to diagnose and manage any underlying conditions that may support the emergence of PVT. Additionally, smoking was found to be a risk factor for PVT, most likely as a result of the harm that smoking causes to endothelial cells [13]. This demonstrates the significance of quitting smoking for PVT patients as well as the potential advantages of early intervention in PVT prevention.

Our study has a few drawbacks. Since the NIS database does not store information about the onset and course of clinical disorders, the temporal sequence of PVT and cirrhosis was not examined. The NIS is not a longitudinal dataset, and it is possible that some recurrent events were counted more than once. Despite this, it makes no difference to our estimate of the overall burden of these disorders. Our large sample size and generalized dataset are significant advantages to be aware of despite these limitations. The large sample size was advantageous, ensuring low confidence intervals, low p-values, and reducing our margin of error. However, it is worth noting that large sample sizes can occasionally skew data, making insignificant differences appear significant. 

## Conclusions

In conclusion, highlights the outcomes of patients with PVT and cirrhosis who also smoke. To reduce the burden of this condition, it is vital to recognize and manage any underlying issues that might either lead to the development of PVT or worsen outcomes. Future research should be aimed at gaining a better understanding of the underlying association between PVT and cirrhosis and developing more effective strategies for the prevention and management of these conditions.
